# Abstract of Meteorological Observations for November, 1853, Made at Philadelphia, Pa.

**Published:** 1854-01

**Authors:** James A. Kirkpatrick


*Abstract of Meteorological Observations for November,* 1853, *made at Philadelphia, Pa. By* Prof. James A. Kirkpatrick.

| 1853. Nov. | Thermometer. Mean. | Range | 2 P. M. Dew point | Barometer. Corrected for Temperature. Mean. | Range. | Prevailing Winds. | General Remarks. |
|---|---|---|---|---|---|---|---|
| | deg. | deg. | deg. | Inches. | Inch. | | |
| 1 | 46.5 | 19 | 39.7 | 30.041 | .110 | S.W. | Clear. |
| 2 | 50.0 | 22 | 44.7 | 29.993 | .077 | W.S.W. | Morn. foggy. aft. and ev. clear. |
| 3 | 52.5 | 17 | 44.7 | 30.092 | .171 | W. | Cloudy. |
| 4 | 45.0 | 14 | 28.7 | 30.305 | .074 | N.W. | Clear. |
| 5 | 41.0 | 12 | 30.7 | 30.307 | .136 | S.W. | do. |
| 6 | 43.5 | 13 | 31.3 | 30.233 | .126 | N.W. | do. |
| 7 | 38.0 | 10 | 26.7 | 30.461 | .044 | N.W. | do. |
| 8 | 44.0 | 17 | 42.7 | 30.256 | .421 | S.W. | Cloudy, ev. rain. |
| 9 | 52.0 | 18 | 52.7 | 29.709 | .261 | W. | do. rain 10 A. M. to 4¾ P. M. |
| 10 | 41.0 | 6 | 25.3 | 30.244 | .230 | N.W. | Clear. |
| 11 | 44.5 | 21 | 41.0 | 30.387 | .120 | (Var.) | Cloudy. |
| 12 | 54.5 | 15 | 57.3 | 30.252 | .226 | E.N.E. | do. drizzling all day. |
| 13 | 59.0 | 10 | 61.7 | 29.569 | .265 | (Var.) | do. do. bar. lowest 29.467, |
| 14 | 48.0 | 12 | 33.0 | 29.691 | .181 | W.N.W. | Clear. [Therm. highest 64°. |
| 15 | 50.5 | 21 | 37.7 | 29.884 | .200 | W. | Cloudy. |
| 16 | 48.0 | 8 | 42.7 | 30.235 | .159 | N.E. | do. ev. rain. |
| 17 | 47.7 | 7 | 49.3 | 30.308 | .025 | N.N.E. | do. aft. and ev. rain. |
| 18 | 50.0 | 10 | 50.3 | 30.284 | .033 | (Var.) | do. |
| 19 | 52.0 | 6 | 52.7 | 30.241 | .035 | S.W. | do. |
| 20 | 55.5 | 17 | 52.3 | 30.095 | .144 | S.W. | do. therm. highest 64°. |
| 21 | 56.0 | 7 | 57.7 | 30.034 | .044 | S.W. | do. showery all day. |
| 22 | 52.7 | 5½ | 51.7 | 30.089 | .020 | S.W. | do. 11 A.M. to 1 P.M. drizzling. |
| 23 | 58.0 | 8 | 55.0 | 29.975 | .085 | S.W. | do. ev. rain. |
| 24 | 40.5 | 21 | 32.7 | 30.063 | .414 | N.W. | M. cloudy, af. & e. clear; *first ice.* |
| 25 | 27.5 | 13 | 31.7 | 30.428 | .025 | (Var.) | Clear. Therm. lowest 21°. |
| 26 | 35.5 | 17 | 34.7 | 30.360 | .088 | W.S.W. | Morn. clear, aft. and ev. cloudy. |
| 27 | 42.0 | 4 | 30.0 | 30.425 | .150 | N.E. | Cloudy. |
| 28 | 42.0 | 8 | 39.0 | 30.482 | .036 | N.N.E. | do. bar. highest 30.502. |
| 29 | 49.0 | 16 | 47.7 | 30.271 | .240 | (Var.) | do. |
| 30 | 47.5 | 11 | 41.3 | 29.993 | .040 | N.W. | Morn. cloudy, ev. clear. |
| Means Nov. 1853 | 47.13 | | 42.22 | 30.157 | | | 2.32 in. rain. |
| Nov. 1852. | 42.15 | | 35.20 | 29.919 | | | 6.05 in. rain. |
| For 2 years. | 44.64 | | 38.71 | 30.038 | | | 4.19 |

Amount of rain fallen during the month, 2.320.

Note.—The observations were made at 7 A. M., 2 P. M., and 9 P. M.

The *Mean Temperature* is the mean of the *lowest* for the day, and the temperature at 2 P. M.

The Dew point is calculated by Mason's hygrometer, as observed at 2 P. M.

